# Tracing the Origins of the Pituitary Adenylate-Cyclase Activating Polypeptide (PACAP)

**DOI:** 10.3389/fnins.2020.00366

**Published:** 2020-05-20

**Authors:** João C. R. Cardoso, Manuel G. Garcia, Deborah M. Power

**Affiliations:** Comparative Molecular and Integrative Biology, Centre of Marine Sciences, University of Algarve, Faro, Portugal

**Keywords:** deuterostomes, early metazoan, evolution, protostomes, neuropeptide, receptor

## Abstract

Pituitary adenylate cyclase activating polypeptide (PACAP) is a well-conserved neuropeptide characteristic of vertebrates. This pluripotent hypothalamic neuropeptide regulates neurotransmitter release, intestinal motility, metabolism, cell division/differentiation, and immunity. In vertebrates, PACAP has a specific receptor (PAC_1_) but it can also activate the Vasoactive Intestinal Peptide receptors (VPAC_1_ and VPAC_2_). The evolution of the vertebrate PACAP ligand – receptor pair has been well-described. In contrast, the situation in invertebrates is much less clear. The PACAP ligand – receptor pair in invertebrates has mainly been studied using heterologous antibodies raised against mammalian peptides. A few partial PACAP cDNA clones sharing >87% aa identity with vertebrate PACAP have been isolated from a cnidarian, several protostomes and tunicates but no gene has been reported. Moreover, current evolutionary models of the peptide and receptors using molecular data from phylogenetically distinct invertebrate species (mostly nematodes and arthropods) suggests the PACAP ligand and receptors are exclusive to vertebrate genomes. A basal deuterostome, the cephalochordate amphioxus (*Branchiostoma floridae*), is the only invertebrate in which elements of a PACAP-like system exists but the peptides and receptor share relatively low sequence conservation with the vertebrate homolog system and are a hybrid with the vertebrate glucagon system. In this study, the evolution of the PACAP system is revisited taking advantage of the burgeoning sequence data (genome and transcriptomes) available for invertebrates to uncover clues about when it first appeared. The results suggest that elements of the PACAP system are absent from protozoans, non-bilaterians, and protostomes and they only emerged after the protostome-deuterostome divergence. PACAP and its receptors appeared in vertebrate genomes and they probably shared a common ancestral origin with the cephalochordate PACAP/GCG-like system which after the genome tetraploidization events that preceded the vertebrate radiation generated the PACAP ligand and receptor pair and also the other members of the Secretin family peptides and their receptors.

## Introduction

The pituitary adenylate cyclase-activating polypeptide (PACAP) is one of the most extensively studied neuropeptides due to its biomedical interest and its well-conserved functions in vertebrates. The first description of PACAP was over 30 years ago when it was identified in extracts of ovine hypothalamus as a factor that stimulated cAMP production in anterior pituitary cells (Miyata et al., 1989, 1990). Since then PACAP has been isolated and characterized in representatives of most of the major vertebrate phyla and has a diversity of functions including the regulation of neurotransmission, vasodilation, intestinal motility, cell proliferation and differentiation and immunity (Sherwood et al., 2000; Vaudry et al., 2000, 2009). Recently PACAP was also described as a potent antimicrobial peptide (AMP) (Starr et al., 2018).

Pituitary adenylate cyclase-activating polypeptide has been linked to several clinical disorders that have highlighted its important role as a neurotransmitter and also its neuroprotective actions (Shioda and Nakamachi, 2015; Maasz et al., 2017; Denes et al., 2019). In the mammalian brain PACAP is most abundant in the hypothalamus from where it is secreted to the pituitary gland but it is also detected in other brain regions such as the telencephalon, cerebellum, and brainstem (Arimura et al., 1991; Ghatei et al., 1993; Hirabayashi et al., 2018; Warfvinge and Edvinsson, 2019). In rat the distribution of the PACAP system is relatively well-characterized and PACAP maps to the parvo- and magnocellular neurons of the paraventricular (PVN) and supraoptic (SON) nuclei of the hypothalamus (Vaudry et al., 2009; Warfvinge and Edvinsson, 2019).

**FIGURE 1:**
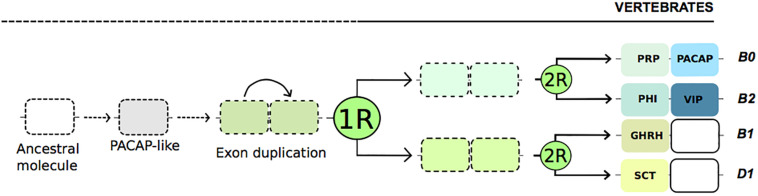
Current evolutionary model for Secretin peptide family members. The exons of the peptide coding region are represented. PACAP-like family members are proposed to have evolved from a common ancestor exon early in the chordate radiation via exon and gene/chromosome duplication events (Sherwood et al., 2000; Cardoso et al., 2007a, 2010; Ng et al., 2012; Hwang et al., 2013). The same ancestral molecule is also suggested to originate the glucagon(GCG)-like peptides but for simplicity this is not represented in the figure. Boxes represent exons and lines introns and the peptide coding exons are indicated by the peptide abbreviation. Dashed lines indicate undefined evolutionary pathways. PACAP and PRP and VIP and PHI share the same gene precursor and GHRH and SCT are encoded by a single exon. The predicted position of each gene block in the Gnathostome Ancestral Genome (GAC) linkage groups are indicated (Hwang et al., 2013). The genome tetraploidization events (1R and 2R) are represented.

Pituitary adenylate cyclase-activating polypeptide is a member of the Secretin brain-gut peptide superfamily. Secretin (SCT) was identified more than 100 years ago by Bayliss and Starling who demonstrated its role in the regulation of pancreatic secretion and “coined the term” *hormone* to describe the mode of action of the factor (Bayliss and Starling, 1902). The SCT superfamily is a group of small peptides that share similarity at the level of their amino acid sequence and structure. In humans the PACAP-like superfamily includes SCT, Vasoactive Intestinal Peptide (VIP), Peptide Histidine Isoleucine (PHI), PACAP-Related Peptide (PRP) and Growth Hormone-Releasing Hormone (GHRH). The glucagon-like peptides (Glucagon, GCG; Glucagon-Like Peptide 1 and 2, GLP 1 and 2; and Glucose-dependent Insulinotropic Peptide; GIP) are also members of the SCT superfamily but they diverged earlier than the other peptide members. All the peptides of the SCT superfamily are proposed to have arisen from a common ancestral gene by exon duplication followed by local duplication and expansion during the two genome tetraploidization events prior to the vertebrate radiation (Figure 1) (Sherwood et al., 2000; Cardoso et al., 2007a, 2010; Ng et al., 2012; Hwang et al., 2013). Vertebrate PRP is encoded by the same gene as PACAP and the peptide VIP is encoded in the same gene precursor as PHI but SCT and GHRH are encoded by specific genes. Within the SCT family of peptides, PACAP shares the highest amino acid sequence resemblance (up to 68%) with VIP, a peptide that was first described as a potent vasodilator in the pig small intestine by Said and Mutt (1970).

In mammals, PACAP is encoded by the *ADCYAP1* gene, which has four exons. Exon 3 of the gene encodes the peptide PRP and exon 4 encodes PACAP and originates two biologically active peptide isoforms (Sherwood et al., 2000; Vaudry et al., 2000, 2009). PACAP-38 is the predominant form and the shorter form, PACAP-27, arises by post-translational processing of PACAP-38 and shares the same N-terminal amino acid (aa) sequence but has a shorter C-terminus (Miyata et al., 1989, 1990; Cox, 1992; Arimura and Shioda, 1995; Sherwood et al., 2000; Vaudry et al., 2000, 2009). In the genomes of mammals and other tetrapods a single *ADCYAP1* gene exists and the peptide it encodes has high sequence conservation between species. In fish a single gene encoding the PACAP peptide precursor that shares high sequence similarity and organization with the tetrapod homolog was identified in the genomes of lamprey, elephant shark, spotted gar, and coelacanth. In contrast, in the teleosts, two PACAP precursor genes *adcyap1a* (protein; Pacapa) and *adcyap1b* (protein; Pacapb), have been isolated and arose from the teleost specific genome duplication event and four mature PACAP peptides (two PACAP-38 and two PACAP-27) are considered to be produced (Cardoso et al., 2007a, 2015; Ng et al., 2012). Analysis across the vertebrates of the genes flanking *ADCYAP1* reveals syntenic genome regions in fish and tetrapods and supports a common evolutionary origin for PACAP (Cardoso et al., 2007b, 2015).

In amphibian and fish (teleost and cartilaginous fish) brain PACAP is abundant and has a similar distribution to that in mammals and is predominantly expressed in the hypothalamic nuclei but also in other brain regions (Valiante et al., 2006; Vaudry et al., 2009). In the zebrafish that has duplicated *adcyap1* genes, transcripts for *adcyap1a* are most expressed in the brain stem and diencephalon, while *adcyap1b* gene transcripts are abundant in the telencephalon and diencephalon (Nakamachi et al., 2019). Although, the distribution of PACAP is relatively well-characterized in fish, few studies have characterized the function of the teleost duplicate PACAPs. The outcome of the studies that exist suggest that the duplicate PACAPs possess similar functions to the mammalian homolog although teleost specific functions are proposed to have also been acquired (Cardoso et al., 2010, 2015).

**FIGURE 2:**
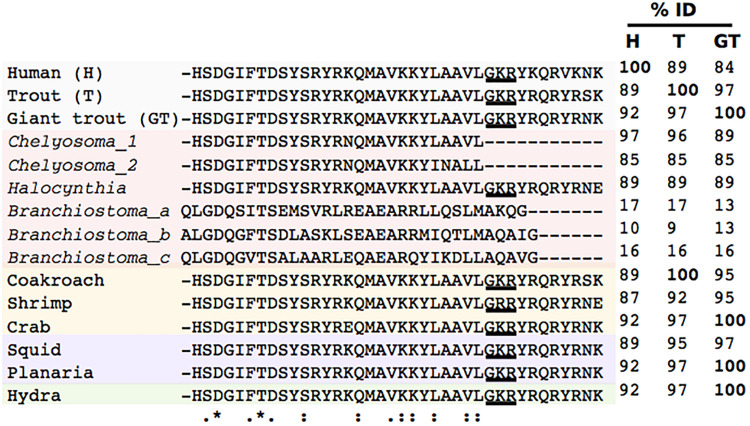
Sequence conservation of the invertebrate PACAP mature peptides. The mature sequence of the invertebrate peptides were extracted and compared with PACAP from the human (P18509) and two Salmonidae fish the river trout (*Salmo trutta*, XM_029756051.1) and the Siberian giant trout (*Hucho taimen*, HAGJ01147357.1) peptide homologs. The post-translational internal cleavage–amidation site (Gly^28^-Lys^29^-Arg^30^) that generates the shortest peptide isoform (PACAP-27) in human, which is predicted in the other peptide sequences is underlined. The percentage of amino acid sequence identity (%ID) for the human (H), river trout (T), and Siberian giant trout (GT) mature PACAP-38 peptides is given. The vertebrate, hydra, protostome and invertebrate deuterostome mature peptide sequences were used to interrogate the protozoan, non-bilaterian, protostome and invertebrate deuterostome genomes and transcriptomes for homologs. Accession numbers of the non-vertebrate peptides are: cockroach (*Periplaneta americana*, AB083652), crab (*Eriocheir japonica*, AB121765), squid (*Sepioteuthis lessoniana*, AB083651), planarian (*Dugesia japonica*, AB083649), and hydra (*Hydra magnipapillata*, AB083650). The tunicate *Chelyosoma productum* (Chelyosoma-1 and Chelyosoma_2, were obtained from McRory and Sherwood, 1997) and the shrimp (*Litopenaeus vannamei*) from Lugo et al. (2013). Complete amino acid conservation is annotated with “*”, partial conservation with “.” and the position of the consensus amino acids conserved in the greatest number of sequences is indicated with “:”.

Homologs of the PACAP system have been predicted in invertebrates and the identified peptides share high sequence conservation with vertebrate PACAP (McRory and Sherwood, 1997; Cardoso et al., 2007a, 2010; Kiss and Pirger, 2013; Lugo et al., 2013; Pirger et al., 2016) (Figure 2). This proposal is based on the isolation of partial cDNAs encoding a PACAP-like peptide from a cnidarian, a few protostomes and tunicates and immunohistochemical studies with heterologous antisera in the 1980 and 1990s (Pirger et al., 2016). Surprisingly, data mining of publicly available invertebrate genomes in the late 2000s, failed to identify sequence homologs of the vertebrate genes (Cardoso et al., 2007a, 2010). Recently, in a cephalochordate a PACAP/GCG-like peptide and receptor were described and their function characterized (On et al., 2015). The predicted cephalochordate peptides and receptor shared low sequence conservation (<17% peptide, <40% receptor) with the vertebrate homologs, which is at odds with the high sequence conservation of the putative peptides of other invertebrate and raises questions about the existing models for peptide/receptor evolution. The advent of next generation sequencing (NGS) and the massive increase in publicly available invertebrate genomes and transcriptomes provides a unique opportunity to trace the evolution of gene families. In this study we revisited the evolution of PACAP by examining past studies and exploring current publicly available genome and transcriptome data for representatives of major non-vertebrate phyla (Protozoans, non-bilaterians, Ecdysozoans, Lophotrochozoan, and invertebrate deuterostomes). The nomenclature for PACAP and its receptors used in this review for the vertebrate species follows the guidelines established by the International Union of Pharmacology (IUPHAR) (Harmar et al., 1998) and the zebrafish nomenclature convention for fish^1^ (Table 1). Nomenclature for urochordate, cephalochordate and protostome follow (McRory and Sherwood, 1997; On et al., 2015; Pirger et al., 2016).

**TABLE 1:** Nomenclature for PACAP and its receptors.

PACAP	PACAP receptor		
Gene/transcripts	Peptide	Gene/transcripts	Protein
**Primate**			
*ADCYAP1*	PACAP	*ADCYAP1R1*	PAC_1_
		*VIPR1*	VPAC_1_
		*VIPR2*	VPAC_2_
**Mammalian (non-primate)**			
*Adcyap1*	PACAP	*Adcyap1R1*	PAC_1_
		*Vipr1*	VPAC_1_
		*Vipr2*	VPAC_2_
**Aves**			
*ADCYAP1*	PACAP	*ADCYAP1R1*	PAC_1_
		*VIPR1*	VPAC_1_
		*VIPR2*	VPAC_2_
**Actinopterygii (non-teleost)**			
*adcyap1*	Pacap	*adcyap1r1*	Pac_1_
		*vipr1*	Vpac_1_
		*vipr2*	Vpac_2_
**Teleost**			
*adcyap1a*	Pacapa	*adcyap1r1a*	Pac_1_a
*adcyap1b*	Pacapb	*adcyap1r1b*	Pac_1_b
		*vipr1a*	Vpac_1_a
		*vipr1b*	Vpac_1_b
		*vipr2a*	Vpac_2_a
		*vipr2b*	Vpac_2_b
**Agnathan**			
*ADCYAP1*	PACAP	*VIPR*	VPAC
**Urochordate**			
*pacap1/pacap2*	PACAP	ni	ni
**Cephalochordate**			
*PACAP/GCG*	PACAP/GCG	*PACAP/GCGR*	PACAP/GCGR
**Protostomes**			
ni	PACAP	ni	ni
**Cnidaria**			
ni	PACAP	ni	ni

*Receptor nomenclature is according to IUPHAR (Harmar et al., 1998) and the ZFIN Zebrafish Nomenclature Convention (https://wiki.zfin.org/). Nomenclature for urochordate, cephalochordate, and protostome follow (On et al., 2015; Pirger et al., 2016). ni, not identified.*

## Pacap Receptors in Vertebrates

The discovery of PACAP in vertebrates was soon followed by the identification of its specific receptor PAC_1_. PACAP also stimulates the activity of the VIP receptors (VPAC1 and VPAC2) and this explains why the two peptides have an overlapping spectrum of physiological activities. The receptors for PACAP have a widespread distribution in the CNS of vertebrates (reviewed by Harmar et al., 2012; Hirabayashi et al., 2018). In the rat brain PAC_1_ is most abundant and has a similar distribution to PACAP and is present in the hypothalamus and non-hypothalamic brain regions (e.g., cerebellum and spinal cord). The expression of the other PACAP receptors is sparse and VPAC_1_ is found in the cerebral cortex and hippocampus and VPAC_2_ in the amygdala, hippocampus, thalamus, and hypothalamus (Hirabayashi et al., 2018). Overall the PACAP system maps to brain regions associated with the stress response, reward seeking and aversive responses (Warfvinge and Edvinsson, 2019).

Pituitary adenylate cyclase activating polypeptide receptors and the receptors for other SCT-like peptides [e.g., parathyroid hormone/parathyroid hormone-related peptide (PTH/PTHrP), calcitonin/calcitonin gene-related peptide (CAL/CGRP), and corticotrophin-releasing hormone (CRH)] form a large family of receptor proteins that belong to the GPCR family B1, also known as Secretin-GPCRs or class II. Family B1 GPCRs possess seven transmembrane domains and a relatively long N-terminus (∼120 amino acids) with six conserved cysteine residue that form three disulphide bridges and create a ligand binding pocket (Harmar, 2001; Couvineau et al., 2012; Harmar et al., 2012). Family B1 receptors share a common origin and emerged prior to the protostome–deuterostome divergence since homologs of the vertebrate members exist in invertebrates (Cardoso et al., 2006, 2014; Hwang et al., 2013). B1 family receptors are suggested to share the same ancestral precursor gene as the adhesion-GPCRs, a group of receptors involved in cell growth, differentiation, and immunity (Springer, 1990; Rosales et al., 1995; Nordström et al., 2009).

Upon receptor binding the PACAP peptide triggers intracellular signal transduction and a biological response. Signaling involves trimeric G-protein complexes that when coupled to the receptor C-terminal domain, stimulate a series of intracellular signaling pathway, which predominately involve, (a) the production of cyclic adenosine monophosphate (cAMP) via the adenylate-cyclase (AC) pathway or (b) the mobilization of the calcium ion (Ca^2+^) pathway involving phospholipase C and inositol 1,4,5-triphosphate (IP3) activity (Rawlings, 1994; Laburthe and Couvineau, 2002; Harmar et al., 2012; Langer, 2012). In teleosts, PACAP receptor number is duplicated in comparison to tetrapods. Six putative PACAP receptor genes (two Pac_1_, *adcyap1r1a* and *adcyap1r1b*; two Vpac_1_, *vipr1a* and *vipr1b* and two Vpac_2_, *vipr2a*, and *vipr2b*) have been characterized and receptor activation triggers similar signaling pathways to those in mammals, birds, and amphibians. The teleost PACAP receptor gene paralogs stimulate similar functions to those of other vertebrates but also acquired specialized functions during the teleost radiation (Cardoso et al., 2004, 2007b, 2015; Roch et al., 2009). In common with the human receptors, interaction of teleost family B1 GPCRs with receptor activity-modifying proteins (RAMPs) (Christopoulos et al., 2003; Archbold et al., 2011; Couvineau and Laburthe, 2011), a class of membrane accessory proteins, can modulate their activity by changing receptor pharmacology (Cardoso et al., 2015).

## The Puzzling Existence of a PACAP Precursor in Invertebrates

Pituitary adenylate cyclase activating polypeptide is proposed to be one of the most well-conserved neuropeptides in the animal kingdom since it has been reported to exist from invertebrates to vertebrates. In invertebrates, putative PACAP-like peptides sharing high sequence identity (>87% aa identity) with the human homolog or identical to the teleost fish peptides have been described (Figure 2). For example, in tunicates, the closest relative to vertebrates (Delsuc et al., 2006), two full length cDNAs (*pacap1* and *pacap2*) encoding PACAP peptides were isolated from the marine disk-top tunicate (*Chelyosoma productum*) (McRory and Sherwood, 1997) and a putative partial PACAP cDNA was isolated from the sea pineapple (*Halocynthia roretzi*) and their deduced peptides were highly identical in sequence to the vertebrate PACAP (Figure 2). In the cnidarian (*Hydra magnipapillata*) and several protostomes partial PACAP cDNA sequences have also been reported. Three arthropods, the crab (*Eriocheir japonica*), the white shrimp (*Litopenaeus vannamei*) and the cockroach (*Periplaneta americana*), a mollusc, the squid (*Sepioteuthis lessoniana*), and the planarian (*Dugesia japonica*) are reported to possess cDNA encoding putative peptides highly identical (>89% aa identity) to the human (Kiss and Pirger, 2013; Lugo et al., 2013) and the trout peptides (>95% aa identity) (Figure 2). A PACAP gene or transcript homolog from invertebrate species with a sequenced genome remains to be convincingly demonstrated (Cardoso et al., 2010).

Furthermore, searches performed in the sea squirt [*Ciona intestinalis*, a.k.a. *Ciona intestinalis type A* (*Ciona robusta*)] genome which has evolutionary proximity with the disk-top tunicate (*Chelyosoma productum*) and sea pineapple (*Halocynthia roretzi*) failed to retrieve a homolog peptide encoding gene (Cardoso et al., 2007a, 2010). Similarly, searches in the genome of an echinoderm, the sea urchin (*Strongylocentrotus purpuratus*) also failed to retrieve a homolog of the vertebrate PACAP gene (Cardoso et al., 2010). Recently, a PACAP/GCG-like peptide gene encoding three putative mature peptides was identified in the genome of the cephalochordate, amphioxus (*Branchiostoma floridae*), the closest extant organism to tunicates. However, in contrast to PACAP in other invertebrates, the amphioxus PACAP/GCG-like peptide had extremely low sequence conservation (<17% aa identity) with the vertebrate PACAP and only a few functionally important amino acid residues for the peptide bioactivity were found (Mirabeau and Joly, 2013; On et al., 2015) (Figure 2).

In the present study we took advantage of the recently released genomes (whole genome shotgun assemblies, WGS), and transcriptomes [Transcriptome Shotgun Assembly (TSA), computationally assembled mRNA sequences from ESTs and raw sequence reads, Supplementary Tables 1, 2] to search for molecular evidence that PACAP emerged early during evolution and was highly conserved from single-celled organisms (protozoans) to invertebrates (non-bilaterian animals and from protostomes and invertebrate deuterostomes) and vertebrates. The conserved mature human and invertebrate PACAP-like peptides were used to screen nucleotide databases for sequence homologs (Figure 2). Despite the limitations caused by the small size of the metazoan PACAP mature peptides (non-specific sequence matches tend to be high), we reasoned that the high degree of sequence conservation between the human and the previously described cnidarian, protostome, and tunicate sequences means that if a homolog exists it should be found using sequence identity searches. Searches for PACAP homologs also included the PACAP/GCG-like peptides recently described in the cephalochordate (On et al., 2015). To favor the identification of short peptide hits with strong similarities the BLAST algorithm was automatically adjusted. The state of the art about PACAP or other SCT-like peptides in single cell organisms to invertebrate deuterostomes was updated in the current study. It was reasoned that characterization of PACAP across phylogenetically distinct non-vertebrate species should reveal the origin and evolution of this important neuropeptide and give clues about function that can contribute to understanding the acquisition of its pleotropic actions in vertebrates. The evolution and phylogeny of the PACAP receptor is also briefly considered as an adjunct to understanding ligand evolution (see section “PACAP Receptors in Invertebrates”).

## PACAP Precursor in Protozoans

Protozoans are a group of free-living single-celled organisms or parasitic microorganisms. The first description of PACAP signaling in a single celled eukaryote was obtained from the free-living ciliate protozoan *Tetrahymena thermophila* a biological and biomedical model commonly used to study avoidance behavior (Hassenzahl et al., 2001; Lucas et al., 2004). *T. thermophila* was described to be repelled by human PACAP-38 leading to the suggestion that a receptor for PACAP exists in *T. thermophila*. Although no putative protozoan PACAP receptor has been identified peptide signaling is proposed to be similar to that in vertebrates and involve the activation of an intracellular G-protein complex which stimulates both the Adenylyl Cyclase and phospholipase C pathways but also the NO/cGMP pathway (Hassenzahl et al., 2001; Lucas et al., 2004). In addition, the pharmacological profile of the putative *T. thermophila* PACAP receptor is suggested to be distinct from the vertebrate homolog as it was activated by the vertebrate receptor antagonists (PACAP 6-27 and PACAP 6-38) (Keedy et al., 2003).

The genome of *T. thermophila* is available (wgs projects: AAGF, AFSS) (Stover, 2006) as is the genome and transcriptome for other members of the superphylum Alveolata (taxid: 33630). The Alveolata are a monophyletic group that include the Ciliophora – aveolates that have short hair-like cilia such as is found in *T. thermophila* and the phylum Apicomplexa – a large phylum of parasitic alveolates that includes the malaria parasite. The results of searches of the genomes and transcriptomes of species from this superphylum in the present study failed to retrieve putative peptide transcripts or genes highly related in sequence to the metazoan PACAP.

## PACAP Precursor in Non-Bilaterians

**FIGURE 3:**
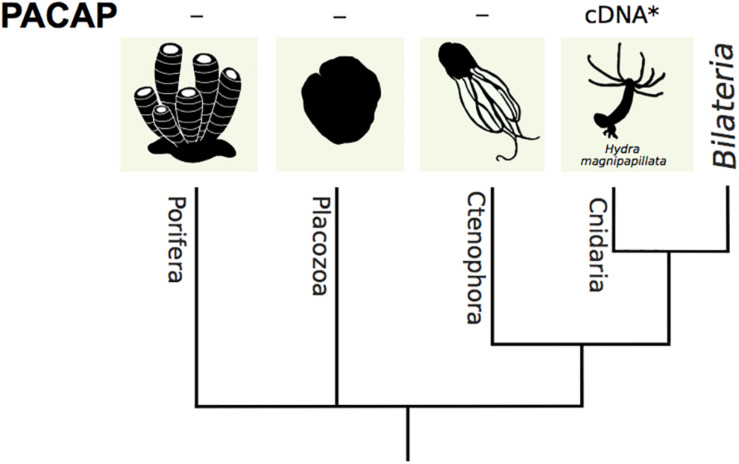
A dendrogram representing the phylogenetic relationship of non-bilaterian phyla. A partial PACAP cDNA was isolated from the *Hydra magnipapillata*. In other non-bilaterian phyla the existence of PACAP remains to be established (-). The evolutionary relationship between the species was based on (Brunet and King, 2017).

The sponges (Porifera), the oldest animal phylum, the comb jellies (Ctenophora), jellyfish and corals (Cnidaria) and plate animals (Placozoa) are four evolutionarily ancient phyla of non-bilaterian animals (Figure 3). They together form the non-bilaterian animals that diverged more than 600 million years ago from the metazoan lineage (Pisani et al., 2015; King and Rokas, 2017). A partial cDNA encoding for a PACAP-like peptide that shares 92% aa identity with human PACAP 38 was isolated and deposited in the NCBI database from the fresh-water polyp *Hydra magnipapillata* (a.k.a. *Hydra vulgaris* or *Hydra attenuata*) (Figure 2) (reviewed in Cardoso et al., 2010; Pirger et al., 2016) and this is the only non-bilaterian PACAP described. No functional or expression data in cnidaria or in any other non-bilaterian animal exists (Figure 3).

Whole body transcriptome data for seven Porifera species, *Amphimedon queenslandica* (GBXN), *Corticium candelabrum* (GAQT), *Cymbastela stipitate* (GHWA); *Haliclona tubifera* (GFAV); *Halisarca caerulea* (GFSI/GFTO/GFTP/GFTQ), *Halisarca dujardini* (HADA), *Sycon coactum* (GAQU) and genome data for two dermosponges (*Amphimedon queenslandica*, ACUQ; *Aplysina aerophoba*, OIVB/OIVD/OIVE/OIVF/OIVG) are available (Supplementary Tables 1, 2). Genome (ABGP) and transcriptome (GFSG) data for the Placozoa *Trichoplax adhaerens* and molecular data for four Ctenophora species, *Beroe ovata* (genome-UOYG), *Mnemiopsis leidyi* (genome-AGCP/TSA project-GFSG), *Pleurobrachia bachei* (genome-AVPN, *Hormiphora californensis*, GGLO) also exist and for Cnidarians an even larger dataset is available including transcriptome and genome assemblies for *Hydra vulgaris* (genome – ACZU/ABRM; TSA projects- GEVZ/GANC/GAOL/GHHG/HAAC/HAAD/GGKF/GGKH) (Chapman et al., 2010). Searches for a potential non-bilaterian PACAP in the present study using the mature sequence of the bilaterian PACAP peptides and cnidarian (peptide and nucleotide sequence) homologs failed to identify a putative transcript or gene.

## PACAP Precursor in Protostomes

The protostomes are the most diverse group of animals and they diverged from the deuterostome lineage approximately 600 million years ago prior to the Cambrian period (Ayala et al., 1998). Two major sister monophyletic protostomian clades that diverged early in evolution exist: (1) the Ecdysozoans and (2) the Lophotrochozoans (Erwin et al., 2011). Studies directed at identifying a homolog of the vertebrate PACAP system are available for both clades.

### Ecdysozoans

**FIGURE 4:**
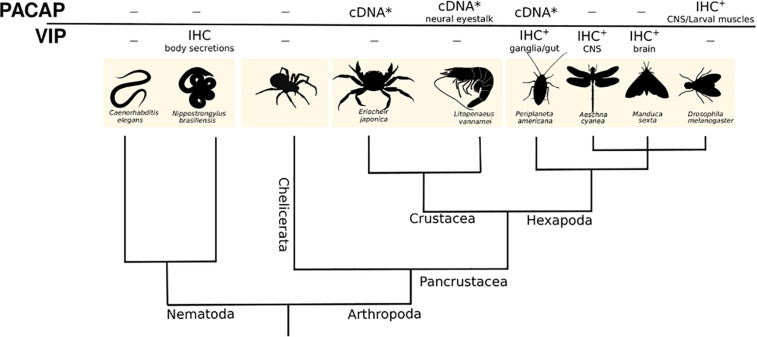
A dendrogram representing the phylogenetic relationships of the main Ecdysozoan phyla. Molecular and expression data (IHC) available for putative PACAP and VIP is presented (El-Salhy et al., 1983; Andries et al., 1984; Andriès et al., 1991). The tissues where putative PACAP or VIP were found are also indicated. - no data available; ^∗^ partial cDNA.

The Ecdysozoa are the largest superphylum of the animal kingdom and include all the arthropods (insects, spiders, and crustaceans), the most diverse and specious animal phyla, the nematodes and several other smaller phyla. This is a morphologically heterogeneous group and includes animals that have a cuticle and grow by molting and over a million species have been described (Telford et al., 2008). Despite their large biodiversity, the basic body plan of Ecdysozoans has been largely conserved and they are either insect-like with a segmented body and jointed appendages or worm-like with an anterior circum-oesophageal nerve ring and a terminal mouth usually found on an introvert (Telford et al., 2008). Ecdysozoans play a central role in the understanding of invertebrate physiology and the nematode *Caenorhabditis elegans* (Consortium, 1998) and the fruit fly *Drosophila melanogaster* genomes (Adams et al., 2000) were the first published animal genomes. In the nematodes there are no reports about the isolation and expression of a PACAP-like system but homologs of the vertebrate VIP and PHI peptides have been detected by dot blot analysis in the excretions/secretions of three parasitic nematodes (*Ascaridia galli*, *Nematodirus battus*, *Nippostrongylus brasiliensis*) (Figure 4) (Foster and Lee, 1996).

In arthropods a PACAP system similar to the vertebrates has been proposed. Antibodies raised against human PACAP-38 were used to detect a peptide homolog in the *Drosophila* central and peripheral nervous systems (Zhong and Peña, 1995). Exposure of larval muscle to the human PACAP-38 peptide modified calcium ion transport (Bhattacharya et al., 2004). Nonetheless, no peptide, transcript or gene for the fruit-fly PACAP or its receptor has been identified, although Western Blot analysis with heterologous antisera led to the suggestion that the putative insect peptide (5.4 kDa) had a similar size to the mammalian homolog (4.5 kDa) (Zhong and Peña, 1995). Subsequently, in *Drosophila* amnesiac peptide was proposed to be the functional homolog of vertebrate PACAP. Sequence similarity between amnesiac and vertebrate PACAP is low but they are proposed to share conserved functions in learning and memory (Feany and Quinn, 1995; Hashimoto et al., 2002). In the insect the American cockroach (*Periplaneta americana*) and in two crustaceans, the crab (*Eriocheir japonica*) and the white shrimp (*Litopenaeus vannamei*), partial PACAP cDNAs have been isolated and the deduced mature peptides are highly identical to the vertebrate peptide (Figures 2, 4) (Lugo et al., 2013; Pirger et al., 2016). No functional studies of PACAP in the cockroach or crab have been described although in the shrimp, innate immunity is boosted (e.g., increased hemocyte number, superoxide dismutase activity, etc.) after bacterial infection in specimens given catfish (*Clarias gariepinus*) recombinant PACAP (Lugo et al., 2013). In arthropods, homologs of other SCT-peptide family members are also suggested to exist and the insect AdipoKinetic Hormones (AKH) are the sequence and function homologs of mammalian GCG and they are also involved in the regulation of food metabolism (sugar homeostasis and mobilization of sugars and lipids from the fat body) (Clynen et al., 2004). In the brain and in the gut muscle layer of the American cockroach immunoreactivity for VIP and PHI has also been detected with heterologous antisera (Figure 4) (Fujita et al., 1981; Iwanaga et al., 1981; Kuramoto et al., 1985).

The Ecdysozoans are the protostome subphylum where the greatest amount of molecular data exists (Supplementary Tables 1, 2). Currently transcriptome assembly data (TSA) for 35 nematodes (free-living and parasitic) and 1410 arthropods (136 Chelicerata, 170 Crustacea, and 1104 Insecta) have been deposited in NCBI (Supplementary Table 1) and were searched in the present study. Whole genome assemblies (wgs) are also available for several representatives of the Nematoda (taxid: 6231) and Arthropoda (taxid: 6656) phyla (Supplementary Table 2). The transcriptome (midgut, GEIF; CNS, GFCQ; whole body, GAWS; testis, GBJC) and genome (PGRX) is available for the American cockroach (*Periplaneta americana*) as is the genome (LQIF) and transcriptome [from precocious and normal juvenile stages, GEFT; eyestalk, Y-organ, hepatopancreas (HAAX, GBZW), fertilized eggs and larvae, GGQO] of a crustacean, the crab (*Eriocheir sinensis*).

Searches in wgs and transcriptomes in the present study failed to yield a homolog gene or transcript of the mature PACAP in representatives of the phylum Arthropoda, subphylum Hexapoda (taxid: 6960, which includes the Insecta), Crustacea (taxid: 6657), Chelicerata (taxid: 6843, which includes the Arachnida) or in the phylum Nematoda (taxid: 6231). In a few species, short positive sequence matches for PACAP were found as part of genes predicted to encode much larger non-PACAP proteins. Interrogation of vertebrate databases with the identified arthropod genes failed to retrieve the PACAP gene or any other homologs of the SCT superfamily suggesting the isolated gene fragments are unlikely to be authentic Ecdysozoan genes for PACAP or other SCT family members.

### Lophotrochozoans

**FIGURE 5:**
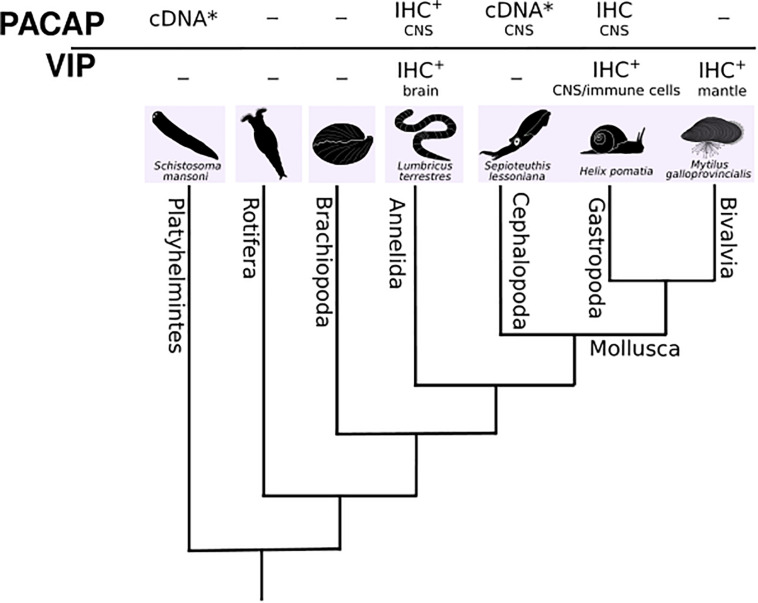
A dendrogram representing the phylogenetic relationships of the main Lophotrochozoan phyla. Available molecular and expression data (IHC) for putative PACAP or VIP is presented (Reglödi et al., 2000; Somogyvári-Vigh et al., 2000; Hernádi et al., 2008; Boros et al., 2010; Pirger et al., 2010a). The tissues where putative PACAP or VIP were found are also indicated. - no data available; * partial cDNA; + heterologous antibody.

The superphylum Lophotrochozoa are the largest group of marine invertebrates and include the greatest number of animal phyla such as molluscs (the second most diverse specious group after the arthropods), annelids and flatworms amongst others (Figure 5) (Kocot, 2016). Far fewer Lophotrochozoan genomes are currently available compared to their sister protostome group, the Ecdysozoans. Nonetheless, comparative genome analysis has revealed that unlike the Ecdysozoans the Lophotrochozoans possess a more similar gene complement and genome organization to deuterostomes (Raible et al., 2005; Miller and Ball, 2009; Takahashi et al., 2009; Simakov et al., 2013). For this reason, it is considered that Lophotrochozoan can contribute to understanding metazoan genome and gene family evolution by creating a link between Ecdysozoans and deuterostomes.

Evidence of a PACAP-like system similar to what exists in vertebrates has been described in lophotrochozoans. In the planarian (*Dugesia japonica*) and in the mollusc squid (*Sepioteuthis lessoniana*) partial cDNAs encoding PACAP-like peptides have been isolated and deposited in the NCBI database and the deduced mature peptides are 92% identical in amino acid sequence to the human peptide (Figure 2) (Cardoso et al., 2007a, 2010; Pirger et al., 2016). In annelids (oligochaeta) and in two gastropod molluscs, the garden snail (*Helix pomatia*) and the pond snail (*Lymnaea stagnalis*) no PACAP precursor has been isolated but results from a series of expression studies have led to suggestions that an active PACAP-like peptide and specific receptor exist (Figure 5) (Pirger et al., 2010a, 2016; Kiss and Pirger, 2013). Using antibodies specific for mammalian PACAP, positive immunoreactivity was detected in the CNS and peripheral organs of adults of three species of annelids (*Lumbricus terrestris*, *Eisenia fetida*, *Lumbricus polyphemus*) and during the embryonic development of the earthworm, *Eisenia fetida* (Reglödi et al., 2000; Boros et al., 2010). PACAP immunoreactivity was also detected in the cerebral ganglia and lip sensory epithelium of the pond snail (Pirger et al., 2010a). In the garden snail, radioimmunoassay using antisera raised against mammalian PACAPs, revealed PACAP-27 and 38 in nervous tissue and peripheral organs and peptide abundance was associated with increased activity of the animals (Hernádi et al., 2008). In both the garden snail and pond snail, PACAP-like peptide fragments were isolated from the brain and two peptide isoforms similar to the vertebrate PACAP-27 and PACAP-38 are proposed to arise from a putative gastropod PACAP gene (Hernádi et al., 2008; Pirger et al., 2010c). Stimulation by mammalian PACAP of cAMP production by pond snail cerebral ganglia homogenates has been proposed to support the existence of a functional PACAP receptor in gastropods, although it has not yet been isolated (Pirger et al., 2010a). In common with mammals, PACAP in snails is proposed to regulate cell proliferation and differentiation and be a neuroendocrine regulator in associative memory (Reglödi et al., 2000; Pirger et al., 2010b, 2016; Kiss and Pirger, 2013; Krajcs et al., 2015) and in gastropods and earthworm PACAP-27 is proposed to be the most abundant form in the CNS (Reglödi et al., 2000; Somogyvári-Vigh et al., 2000; Boros et al., 2008; Hernádi et al., 2008; Pirger et al., 2010a).

Other homologs of the vertebrate SCT peptide family have also been detected in the lophotrochozoans (annelids, molluscs, and platyhelminths) by immunohistochemistry using heterologous antisera (Figure 5) (Cardoso et al., 2010). In annelids, a sister clade of molluscs, immunoreactive PACAP-like peptides and receptors were identified in the CNS and peripheral nervous system (PNS) (Reglödi et al., 2000; Molnar et al., 2006; Boros et al., 2008, 2010; Varhalmi et al., 2008). Similarly, in the bivalve mollusc the Mediterranean mussel (*Mytilus galloprovincialis*), immunoreactivity for VIP was detected in the mantle and in gastropod molluscs, VIP-like molecules were detected in the nervous system of the sea hare (*Aplysia kurodai*) and land snail (*Helix pomatia*) and in innate immune cells of two freshwater snails (*Planorbarius corneus* and *Viviparus ater*) (Kuramoto et al., 1985; Ottavianil and Cossarizza, 1990; Ottaviani et al., 1992; Kaufmann et al., 1995; Licata et al., 2003). In the nervous system of the pond snail (*Lymnaea stagnalis*) two VIP immunoreactive neurons were detected (Schot et al., 1981). Immunoreactive GCG/GLP and SCT was also detected in immune cells of the two freshwater snails (Ottavianil and Cossarizza, 1990; Ottaviani et al., 1992). In annelids, VIP-like positive cells were detected in the CNS of the leech (*Hirudo medicinalis*), earthworm (*Lumbricus terrestres*), oligochaete (*Nereis diversicolor*) (Sundler et al., 1977; Osborne et al., 1982), and planarian (*Schistosoma mansoni*) (Gustafsson, 1987).

For the major lophotrochozoan phyla assembled tissue transcriptomes (TSA) and genomes (wgs, Annelida, taxid: 6340; Mollusca, taxid: 6447 (Bivalvia, taxid: 6544; Gastropoda, taxid: 6448; Cephalopoda, taxid: 6605); Rotifera, taxid: 10190; Brachiopoda, taxid: 7568; Platyhelminthes, taxid: 6157) are available (Supplementary Tables 1, 2). These include a transcriptome and genome for the planarian *Dugesia japonica* (transcriptomes – GFJY, GALW, IAAB, genome- MQRL) and a transcriptome of the sucker ring tissue of the squid *Sepioteuthis lessoniana* (transcriptome – GBGT). Assembled transcriptomes of 21 Annelids, 10 Brachiopods, 41 Platyhelminthes, 9 Rotifers, and 138 molluscs (68 gastropods, 44 bivalves, 26 cephalopods) are also available.

Searches in wgs and transcriptomes of lophotrochozoa in the present study using the mature conserved metazoan and the cephalochordate PACAPs as bait failed to retrieve sequence matches in the majority of the transcriptomes and genomes analyzed, including the planarian and squid, which were previously proposed to possess PACAP. The short sequence hits identified were further analyzed by using them to search the human genome. However, they failed to retrieve PACAP or any other SCT family member. In summary, searches in lophotrochozoan transcriptomes and genomes failed to identify transcripts or genes that shared high sequence identity with human PACAP or other members of the SCT superfamily.

## PACAP Precursor in Invertebrate Deuterostomes

**FIGURE 6:**
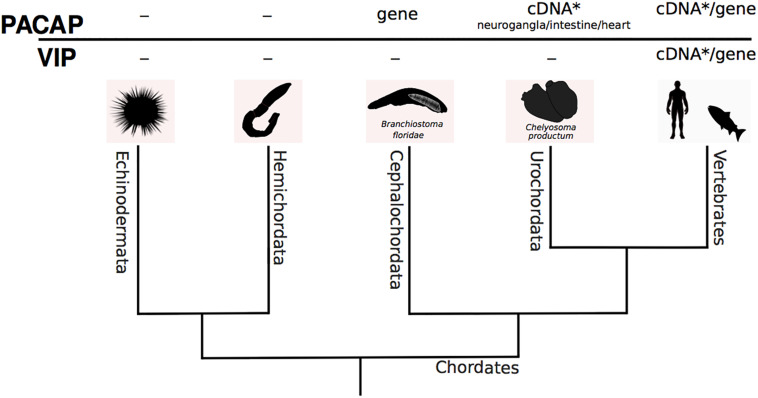
A dendrogram representing the phylogenetic relationship of the invertebrate deuterostome phyla. Available molecular and expression data (PCR and IHC) for putative PACAP and VIP is presented (McRory and Sherwood, 1997). The tissues where putative PACAP or VIP were found are indicated. - no data available; * full-length cDNA.

**FIGURE 7:**
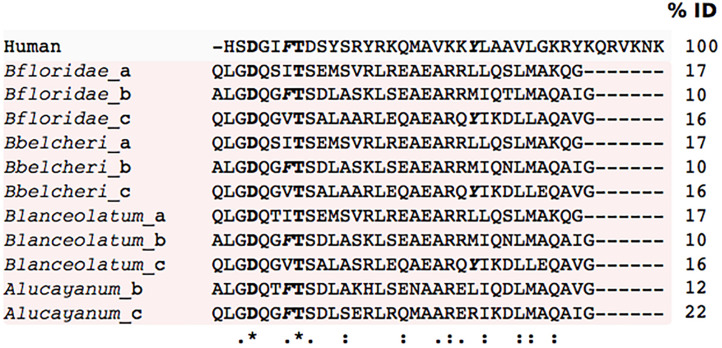
Multiple sequence alignment of the cephalochordate PACAP-like peptides. Comparison of the human mature peptide sequence with the predicted cephalochordate PACAP-like peptides. The percentage of identity (%ID) with the human mature PACAP-38 peptide is given. Complete residue conservation is annotated with a “*” and highlighted in bold, partially conserved residues are denoted by “.” and the position of the consensus amino acids present in the greatest number of sequences are indicated with “:”. Residues in the vertebrate peptides important for receptor activation are annotated in bold and italics.

The invertebrate deuterostomes include the echinoderms, hemichordates, and chordates (urochordates and cephalochordates) and they are all marine animals (Figure 6). Of all the invertebrates, the invertebrate deuterostomes genomes are proposed to be most like the vertebrate genomes. Furthermore, since the invertebrate deuterostomes did not experience a genome tetraploidization they possess a single copy of the gene homologs present as multiple gene copies in vertebrates (Nakatani et al., 2007; Putnam et al., 2008). The invertebrate deuterostomes are regarded as an important link between the protostome–deuterostome ancestor and vertebrates and can provide relevant insight into vertebrate gene family origin and evolution. PACAP precursors have been isolated in tunicates and cephalochordates and the tunicate deduced mature peptides are highly similar to the vertebrate peptides while the cephalochordate peptides are poorly conserved (Figure 2). Other members of the SCT family of peptides have been identified by immunohistochemistry (IHC) in the cerebral ganglion and digestive system of two tunicates (*Ciona intestinalis* and *Styela plicata*) (Pestarino, 1990) and in the digestive tract of a cephalochordate, the common lancelet (*Branchiostomata lanceolatum*) (Reinecke, 1981) (Figure 7). This suggests that a similar gene repertoire to the human SCT family is present in invertebrate deuterostomes and this supports the notion that the gene family may have emerged just prior to the vertebrate radiation.

Two *pacap* transcripts from the disk-top tunicate (*Chelyosoma productum*) encode two PACAP-27 mature peptides, that share 96 and 85% aa sequence identity with human PACAP (McRory and Sherwood, 1997). Tunicate *pacap1* is highly expressed in the neuro ganglia and *pacap2* has a widespread tissue distribution and is also present in the gonad/digestive gland and intestine (McRory and Sherwood, 1997). In the cephalochordate amphioxus (*Branchiostoma floridae*) a single PACAP-like gene and a functional receptor have been characterized (Mirabeau and Joly, 2013; On et al., 2015). The organization of the cephalochordate peptide precursor differs from that of the tunicate and it produces three PACAP-like peptides, bfPACAP/GCGa, bfPACAP/GCGb, bfPACAP/GCGc (32 to 33 aa in length) that share low sequence identity (10–15% aa) with human PACAP and are proposed to have co-evolved with the GCG-peptides (Mirabeau and Joly, 2013). The cephalochordate PACAP/GCG receptor (bf95) also shares relatively low sequence similarity with the human PAC_1_ (On et al., 2015).

Transcriptome (TSA) data available for four tunicates (*Ciona intestinalis*, GBKV; *Ciona savignyi*, GGEI; *Oikopleura* dioica, GCJN and *Salpa thompsoni*, GFCC), two cephalochordates (*Asymmetron lucayanum*, GESY/GETC and *Branchiostoma floridae*, GESZ/GETA/GAMX), a hemichordate (*Ptychodera flava*, GDGM) and 40 echinoderms was investigated in the present study (Supplementary Table 1). Searches in tunicate and hemichordate transcriptomes for PACAP using as the bait the protostome and deuterostome mature PACAPs failed to identify homologs in tunicates, hemichordates, or echinoderms. PACAP homolog sequences were found in searches of cephalochordate transcriptomes. In the Bahama lancelet (*Asymmetron lucayanum*) four potential PACAP homologs with an identical sequence (GESY01044927.1, GESY01044926.1 GESY01044925.1 GESY01044923.1) were retrieved. Putative PACAP homolog sequences were also retrieved from the Florida lancelet (*Branchiostoma floridae*). Further analysis of the isolated transcripts revealed that in both species they correspond to an alternative splice form of the previously published PACAP/GCG transcript (XP_002608413.1). The transcripts identified in the present study lacked the putative bfPACAP/GCGa peptide predicted to occur at the N-terminus of the protein precursor (On et al., 2015). The Florida lancelet transcripts are 100% identical to the PACAP/GCGb and PACAP/GCGc previously described (Mirabeau and Joly, 2013; On et al., 2015) and share 72 and 66% sequence identity, respectively with the predicted PACAP peptides in the Bahama lancelet (Figure 7).

To identify the putative origin of the basal deuterostome PACAP, whole genome assemblies (wgs) for tunicates, hemichordates, echinoderms, and cephalochordates were screened (Supplementary Table 2). In Tunicata (taxid: 7712), Hemichordata (taxid: 10219), and Echinodermata (taxid: 7586) genomes no gene homolog of human PACAP was identified. In cephalochordates (Cephalochordata, taxid: 7735) the PACAP/GCG-like gene was retrieved from the Florida lancelet, Belcher’s lancelet (*Branchiostoma belcheri*, three genome assemblies), common lancelet (*Branchiostoma lanceolatum)* and Bahama lancelet. The deduced cephalochordate peptides shared very low sequence identity with human PACAP and considering all isoforms (a-c) only two amino acid residues were conserved (Figure 7). However, amino acid residues important for receptor binding in human PACAP, Phe^6^ and Tyr^22^ (Sun et al., 2007; Bourgault et al., 2009; Dejda et al., 2011) were conserved in the cephalochordate PACAP/GCG_b and PACAP/GCG_c peptides, respectively (Figure 7). Overall our searches in invertebrate deuterostomes failed to identify sequence homologs of either the human or previously reported tunicate PACAP (McRory and Sherwood, 1997). However, transcripts and genes for cephalochordate PACAP/GCG-like peptides were identified, suggesting that a PACAP-like gene emerged in the lineage giving origin to cephalochordates.

## PACAP Receptors in Invertebrates

Searches for SCT family peptides in invertebrates has frequently been accompanied by searches for the cognate receptors as additional proof that the system exists. In vertebrates’ receptors that are activated by PACAP are member of family B1 GPCRs. To date the only invertebrate PACAP/GCG-like receptor (bf95) isolated and functionally characterized is from the cephalochordate, amphioxus (*Branchiostoma floridae*) but this receptor shares poor sequence similarity (37% aa identity) with the vertebrate PAC_1_ but when it is activated it triggers intracellular signaling processes similar to the mammalian homolog (On et al., 2015). To provide evidence supporting the existence of a non-vertebrate homolog of vertebrate PAC_1_ and to further understand how the peptide-receptor system emerged we searched for putative receptor sequence homologs in representative species of major invertebrate phyla and compared receptor evolution to that of other family B1 GPCRs.

In protostomes, a PACAP-like receptor similar to that in vertebrates was previously predicted in an annelid, *Eisenia fetida* and in the gastropods, *Lymnaea stagnalis* and *Helix pomatia* based on protein detection with heterologous antisera raised against the homolog mammalian receptor. In the earthworm (*Eisenia fetida*) PAC_1_-like immunoreactivity was detected in adult CNS and embryos and the protein was estimated to be 50 kDa and to have a similar organization to the vertebrate homolog (Boros et al., 2010). In the gastropod, abundant PAC_1_-like immunoreactivity was found in both the CNS and peripheral nervous system of the snail *Helix pomatia* and human PACAP-27 and PACAP-38 shown to elicit a response in neurons expressing the receptors (Hernádi et al., 2008). In the nervous system of the snail, *Lymnaea stagnalis*, human PACAP-38 increased cAMP levels (Pirger et al., 2010b). In insects a PACAP receptor has not been isolated although there are studies that suggest a functional receptor may exist.

A study aimed at characterizing G-protein-coupled neurotransmission using an insect “learning model” rutabaga-type *Drosophila* mutants that lack the type I Ca(2+)/CaM-dependent adenylyl cyclase (AC) gene revealed that vertebrate PACAP-38 stimulates synaptic currents through the coactivation of the Ras/Raf and Rutabaga-adenylyl cyclase pathways (Zhong, 1995). Furthermore, human PACAP activates the receptor for insect PDF when co-expressed with Neurofibromatosis 1 (NF1) protein that potentiates PDF action by coupling to AC (Mertens et al., 2005). PDFR and PAC_1_ are both members of family B1 GPCRs and share some structural resemblance although PDFR is exclusively found in invertebrates (Cardoso et al., 2014). The insect *maxadilan* peptide, which is abundant in the saliva of the sand fly (*Lutzomyia longipalpis*), the vector of leishmaniasis, activates human PAC_1_. Despite low sequence similarity *maxadilan* shares similar functions to PACAP and in vertebrates it elicits vasodilation and modifies the secretion of pro-inflammatory cytokines by macrophages (Bozza et al., 1998; Soares et al., 1998). This suggests that in insects there is promiscuity between peptide ligand–receptor pairs and that this plasticity has been used by organisms to advantageously modulate host physiology via the ancient GPCR system. This idea is reinforced by the recent demonstration that human and fish SCT family peptides can modulate the physiology of the mosquito vector of malaria when provided to animals in an artificial meal (Marques et al., 2018). Furthermore, exposure to human GLP 2 peptide (GCG-peptide member) significantly increased vitellogenin expression, mosquito egg production and offspring fitness although if this was due to the activation of mosquito GPCRs was not established (Marques et al., 2018).

**FIGURE 8:**
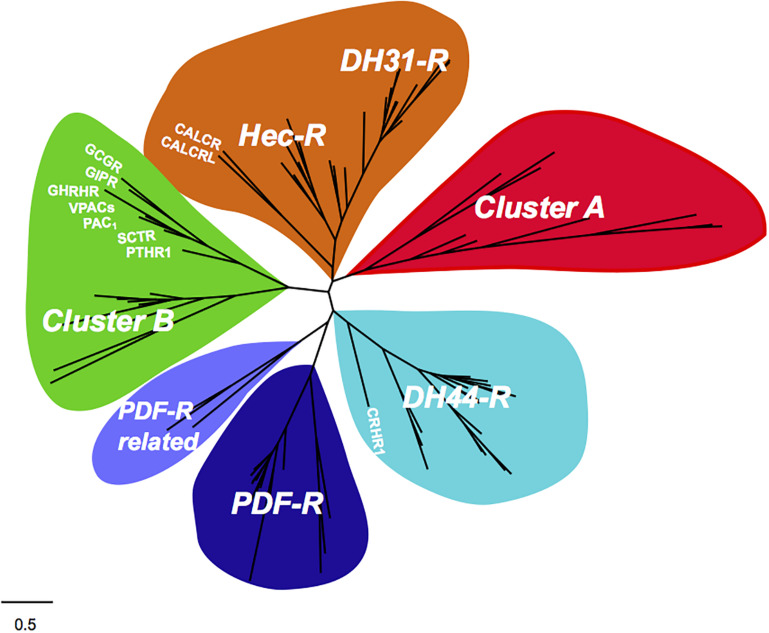
Evolutionary relationships of the nematode and arthropod family B1 GPCRs with the human homologs. The six nematode and arthropod receptor subfamilies are represented in different colors and the human gene homologs are indicated. The phylogenetic tree was modified from Cardoso et al. (2014).

**FIGURE 9:**
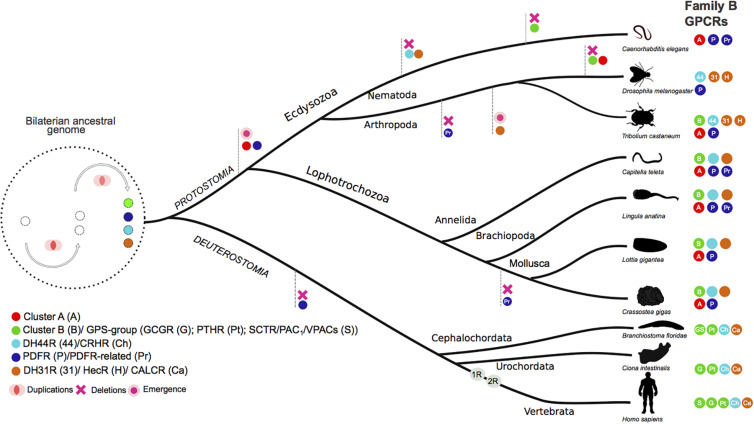
Proposed evolutionary model for the Ecdysozoans and Lophotrochozoan family B1 GPCRs. The main metazoan receptor subfamily gene clusters are represented by full colored circles according to their proposed common origin in the bilaterian ancestral genome. Four genes precursors for family B GPCR subfamilies arouse from gene duplication events in the bilaterian ancestral genome. These genes subsequently evolved under distinct evolutionary pressures in the protostome and deuterostome lineages (for more details see Cardoso et al., 2014). Species-specific gene duplications/deletions within each receptor family are not represented. The two rounds of genome duplication (1R and 2R) in the deuterostome radiation are represented. The phylogeny of Cluster B is represented in Supplementary Figure 1. The rest of the data was obtained from Cardoso et al. (2006; 2010, unpublished; On et al., 2015). The figure was adapted from Cardoso et al. (2014) and is not drawn to scale.

In arthropods, DH31R and DH44R are the sequence homologs of the vertebrate CALCR and CRHRs but invertebrate genomes contain a larger family B1 GPCR receptor gene repertoire most of which are orphans. Recently searches in nematode and arthropod genomes revealed that six main B1 GPCRs subfamilies exist and that they evolved under lineage and species-specific pressure (Cardoso et al., 2014) (Figure 8). In addition to the DH31R and DH44R subfamilies the nematode and arthropod genomes also possess receptors for the peptide PDF (PDFR) and for the recently identified PDFR-related, Cluster A and Cluster B (Cardoso et al., 2014). The PDFR, the PDFR-related and Cluster A have no homolog genes in vertebrates but Cluster B receptors are considered to be the orthologous of vertebrate PAC_1_/VPAC_1_, GCGR, and PTHR with which they probably share a common evolutionary origin (Cardoso et al., 2014) (Figure 9). Receptor members of Cluster B, which are most similar to the vertebrate PAC_1_, have been lost from the genomes of Diptera (*Drosophila melanogaster* and mosquitoes) and from the nematode, *C. elegans*, although the receptor genes persisted in other arthropods as well as in the genome of a parasitic nematode suggesting that they evolved under distinct pressures potentially driven by specific chromosome rearrangements (Cardoso et al., 2014). Considering the nematode and arthropod receptors it has been hypothesized that at least four ancestral family B1 GPCR genes emerged early in the metazoan radiation and underwent distinct evolutionary trajectories after the protostome-deuterostome split (Figure 9).

Nonetheless, in other protostomes, family B1 GPCR genes are mostly unknown in lophotrochozoan which are suggested to have a more similar gene repertoire to vertebrates than arthropods and nematodes (Simakov et al., 2013; Cardoso et al., 2016). Searches for putative sequences related to the vertebrate PAC_1_ in Lophotrochozoan genomes (Molluscs, Annelids, Brachiopod) only retrieved members of the Cluster B subfamily and no direct sequence homologs of vertebrate PAC_1_ were found (Figure 9 and Supplementary Figure 1). Our phylogenetic tree topology confirms that the protostome receptors of Cluster B are the most similar in sequence to the vertebrate GCG/PTH/PACAP receptors. In addition, homologs of the previously identified nematode and arthropod B1 GPCR subfamilies were found and the previously proposed evolutionary model was confirmed. The cephalochordate PACAP/GCGR-like gene diverged prior to the vertebrate *ADCYAP1R1* and *GCGR* genes confirming its identity but no gene homolog was identified in tunicate genomes. In contrast, in the *Ciona* genome two sequence homologs of the vertebrate GCGR-subfamily were retrieved, but a putative PACAP receptor gene was absent (Supplementary Figure 1).

## Conclusion

**FIGURE 10:**
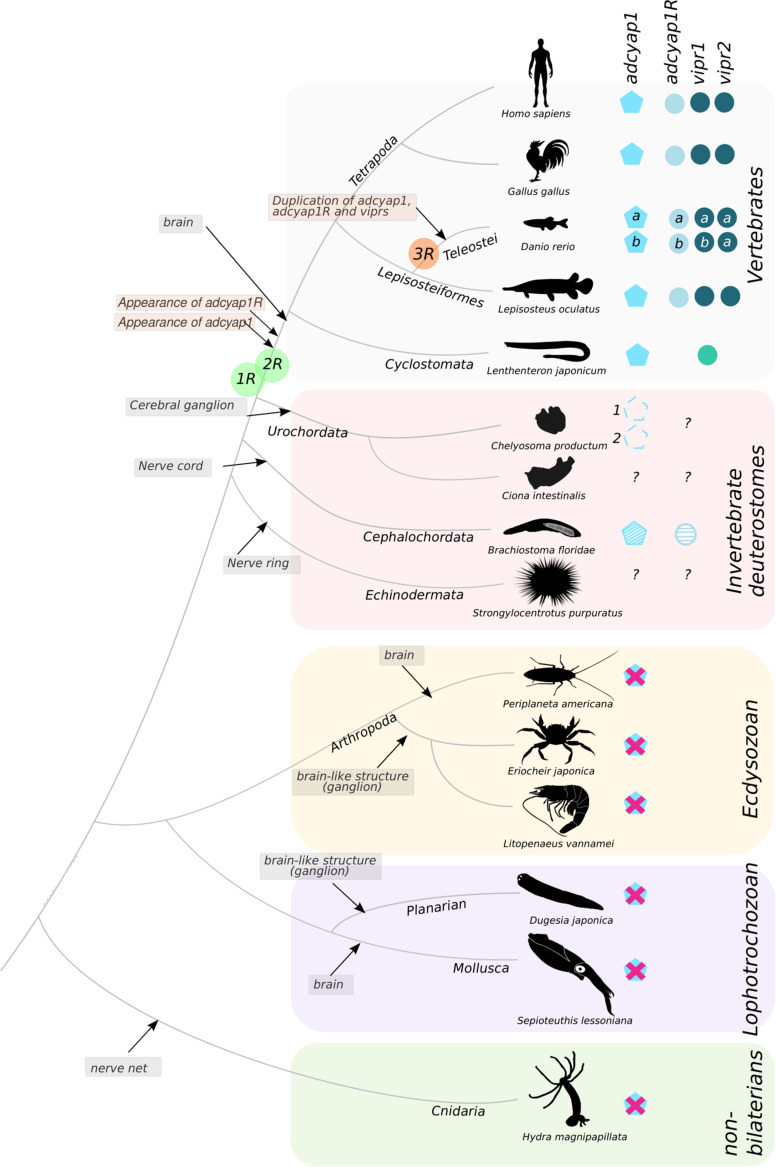
Molecular data for PACAP and PACAP receptors in metazoan. The peptide is represented by pentagons and the receptors by circles. The major events associated with gene family evolution are indicated. In lamprey, a *adcyap1* peptide gene precursor and *adcyap1R1/vipr1* receptor gene were isolated (Ng et al., 2012). In the tunicate *Chelyosoma productum* two PACAP precursors (*pacap1* and *pacap2*) were isolated but their existence remains to be confirmed, as do the PACAP precursors in the related species, *Ciona intestinalis* and in the scheme are represented by a dashed lined pentagon (McRory and Sherwood, 1997; Cardoso et al., 2010). The cephalochordate PACAP-like peptides and receptors are represented by striped pentagons and circles, respectively, as they are hybrids of the vertebrate PACAP/GCG peptide and receptor system (On et al., 2015). The genome duplication events that occurred earlier during the vertebrate radiation (1R, 2R) and the teleost specific (3R) are also annotated. The teleost peptide and receptor genes that resulted from 3R are represented by a and b. The major evolutionary events that explain existing molecular data are mapped with boxes with a flesh colored background. The main events associated with the appearance of the metazoan nervous system are also represented. The evolutionary relationship between the species represented was based on (Brunet and King, 2017). A cross (X) means not found in molecular databases (TSA and WGS) and likely to be absent in the species represented. ?- unknown existence.

No evidence for a highly conserved PACAP system or any other member of the SCT superfamily outside the vertebrate clade was found in our study. Molecular searches of numerous representatives of major non-vertebrate phyla failed to identify a putative gene or transcript of the PACAP peptide precursors previously reported in the tunicate (*Chelyosoma productum* and *Halocynthia roretzi*), crab (*Eriocheir japonica*), shrimp (*Litopenaeus vannamei*), cockroach (*Periplaneta americana*), squid (*Sepioteuthis lessoniana*), planarian (*Dugesia japonica*), or cnidarian (*Hydra vulgaris*) (Figure 10). Our searches only revealed genes and transcript homologs of the cephalochordate (*Branchiostoma floridae*) PACAP/GCG-like precursor in other related cephalochordate species. Similarly, in protostomes only Cluster B receptor genes were identified and phylogenetic analysis indicates they probably shared a common ancestral origin with the vertebrate *ADCYAP1R1* gene but also with *GCGR* and *PTHR* subfamily genes.

The origin of the previously reported protostome cDNAs encoding a peptide highly similar to vertebrate PACAP is difficult to explain. The peptide and nucleotide sequences of protostome PACAP overlap totally (100% aa and nucleotide identity) with PACAP-family precursors from salmoniformes (*Salmo trutta*, *Oncorhynchus nerka*, and *Oncorhynchus tshawytscha*) (Figure 2 and Supplementary Table 3) suggesting that they may be artifacts. The existence of PACAP genes/transcripts in urochordates remains unresolved as sequence homologs (peptides or nucleotides) of the isolated *Chelyosoma productum* PACAP precursors (McRory and Sherwood, 1997) were not identified in other urochordates or invertebrate deuterostomes. We propose a checklist for establishing the validity of cDNA encoding PACAP or other regulatory peptides. Specifically, proof should be provided at the level of gene, protein and function for confirming the veracity of gene/cDNA/peptide identity. If a genome is available the cDNA should be mapped and the gene confirmed by PCR; codon usage bias should be considered and clustering in phylogenetic analysis is expected generally to follow accepted models for phyla relatedness; independent confirmation from a lab focusing only on non-vertebrates would be encouraged. At the level of proteins, it should be possible to isolate PACAP-like peptides and then confirm by *de novo* peptide sequencing the identity. Finally, at the level of function vertebrate peptides may be used in invertebrates but proof from CRISPR-Cas9 or interfering RNA of activity ablation is essential along with complimentary experiments with the receptors in the case of neuropeptides. While it may not always be possible to gather strong functional proof in non-model organisms at least robust analysis at the level of nucleic acids and proteins is an important step for identification.

Receptors for family B1 GPCRs emerged much earlier than the ligands of the SCT superfamily of peptides as we identified homologs of the vertebrate CALCR and CRHR in protostomes. The Cluster B receptors are closest to the vertebrate PACAP receptors but based on the molecular evidence gathered we propose that the *ADCYAP1R1* gene only appeared during the vertebrate radiation at a similar time to the *ADCYAP1* gene (Figure 10). In the most ancient extant vertebrate representative, the lamprey and hagfish (cyclostomes) an *ADCYAP1* gene and two *VIPR* genes were identified but an *ADCYAP1R1* gene was absent. Nonetheless, it is not possible to rule out that it never existed in their genomes, which are highly modified due to the independent gene duplications/deletions and genome rearrangements that occurred after the gnathostome divergence (Ng et al., 2012). The results of the previous protostome IHC studies using heterologous antibodies raised against human peptides and receptors are puzzling and the positive signals obtained may be due to interactions with other related GPCRs or unrelated proteins that bear some similarity with human PACAP receptors. We propose that strict procedures should be followed for IHC with heterologous antisera to minimize cross-reactivity or low specificity interactions. This should involve, (a) the identification of the protein/peptide sequence used to raise homologous antisera and confirmation of peptide existence in the experimental model by searches against genome/transcriptome data, (b) a battery of control assays to check for antisera specificity using recombinant proteins (if they exist) or peptides used to raise the antisera, antisera pre-absorption studies, staining reactions on multiple individuals and sections to confirm if the general staining pattern is conserved and Western blots using protein extracts of the species being studied, with and without peptide pre-absorption of antisera to check if one principle protein of the predicted size is detected.

The explanation for the effects caused by exposure of protostome tissues to human PACAP peptides is uncertain but may be due to low specificity ligand–receptor interactions that have previously been reported for heterologous peptides (Marques et al., 2018). In summary, based on our in-depth molecular study we propose that the *ADCYAP1* gene appeared in vertebrates and probably shared a common origin with the cephalochordate PACAP/GCG-like gene. The ancestral PACAP/GCG-like gene probably expanded during the tetraploidization events preceding the vertebrate radiation (1R and 2R) and generated the *ADCYAP1* gene and other members of the SCT-family peptides (Figure 10).

Comparative endocrinology is crucial for clinical/pharmacology research and identification of homolog systems is important to understand the endocrine peptide-receptor function and regulation (Cardoso and Larhammar, 2014). In addition, it also provides insights into how function has drifted and changed during evolution and facilitates the discovery of novel ligand–receptor pairs. In the future we proposed that characterization of the Cluster B receptor–ligand pair may provide important clues about the function of the PACAP-like system in metazoans and how this is linked with the acquisition of a nervous system and neuropeptide signaling. Given the multifunctional role of PACAP in vertebrates, characterization of such molecules would provide novel insights into the regulatory role of the PACAP-system and studies of much broader scope particularly in under represented phyla would contribute to the development of more robust evolutionary models to explain the emergence and persistence of PACAP and how its pleotropic role was acquired during evolution.

## Data Availability Statement

The datasets analyzed in this study can be found at https://www.ncbi.nlm.nih.gov. All TSA and WGS databases enquired (accession numbers and bioprojects) are listed in Supplementary Tables 1 (TSA) and 2 (WGS). Accession numbers for all sequences used are cited in the paper and when not available the study where it was described is indicated in the text.

## Author Contributions

DP and JC conceived and planned the study, analyzed and integrated the datasets, and wrote the manuscript. JC and MG performed the bioinformatic analysis searches and prepared the figures. All authors critically read the manuscript.

## Conflict of Interest

The authors declare that the research was conducted in the absence of any commercial or financial relationships that could be construed as a potential conflict of interest.

## Footnotes

1 https://wiki.zfin.org/

## Supplementary Material

FIGURE S1 Phylogenetic trees of the invertebrate Cluster B receptors and the deuterostomes PAC_1_, GCGR, and PTHR members. The consensus phylogenetic trees were obtained **(A)** according to the Bayesian inference (BI) and **(B)** the maximum likelihood (ML) methods using has input an edited multiple sequence alignment of the predicted receptor protein sequences obtained using the MUSCLE algorithm (Edgar, 2004) available from Aliview platform 1.18 (Larsson, 2014). BI phylogenetic reconstruction was performed using MrBayes 3.2.6 (Ronquist et al., 2012) in the CIPRES Science Gateway V. 3.3 (http://www.phylo. org) and ML tree was performed using the PhyML3.0 program available from the ATGC platform (http://www.atgc-montpellier.fr/phyml/). The BI and ML trees were constructed with an LG substitution model according to the Akaike information criterion (Lefort et al., 2017) and BI used 1,000,000 generation sampling probability values to support tree branching and ML statistical branch support was 100 bootstrap replicates and >50 are mapped. The cephalochordate sequences are highlighted in pink and were obtained from On et al. (2015). The Arthropod receptor sequences are highlighted in yellow and were obtained from Cardoso et al. (2014) and the Lophotrochozoan sequences (highlighted in purple) were obtained by querying the metazoan ENSEMBLE GENOMES (http://metazoa. ensembl.org/index.html) database using the human receptors. Accession numbers of the invertebrate sequences are indicated in the tree. Accession numbers of human receptors are: CALCR, NP_001733.1; CALCRL, NP_005786.1; CRHR2, NP_001874.2; CRHR1, NP_004373.2; PTH1R, NP_000307.1; PTH2R, NP_005039.1; VPAC_1_, NP_004615.2; PAC_1_, NP_001109.2; VPAC2, NP_003373.2; GCGR, NP_000151.1; GLP1R, NP_002053.3. The tree was rooted using the human CALC/CALCRL and CRHR1/CRHR2 branches.

TABLE S1 List of non-vertebrate transcriptome databases and their tissue of origin that were searched for PACAP transcripts. Available data from NCBI, November 2019. TSA databases that were available to search at NCBI are listed. Bioproject numbers are indicate.

TABLE S2 List of whole genome assemblies that were searched for non-vertebrate PACAP genes. Available data from NCBI, November 2019. Bioproject numbers are indicate.

TABLE S3 List of the hydra, protostome and tunicate PACAP nucleotide top five hits against the NCBI database **(A)** and Salmoniformes (taxid: 8006) transcriptomes (TSA) **(B)**. The e-values (e value) and percent of identity (%ID) are given ^∗^ Nucleotide sequence not available.
