# Corrigendum: Tracing the Origins of the Pituitary Adenylate-Cyclase Activating Polypeptide (PACAP)

**DOI:** 10.3389/fnins.2020.00801

**Published:** 2020-08-18

**Authors:** João C. R. Cardoso, Manuel G. Garcia, Deborah M. Power

**Affiliations:** Comparative Molecular and Integrative Biology, Centre of Marine Sciences, University of Algarve, Faro, Portugal

**Keywords:** deuterostomes, early metazoan, evolution, protostomes, neuropeptide, receptor

In the original article, there was a mistake in the legend for Table 1 as published. The table describes nomenclature for both vertebrates and non-vertebrates.

In the original article, there was a mistake in Table 1 as published. PACAP receptor nomenclature for teleost and Actinopterygii (non-teleost) has errors. The Agnathan PACAP Receptor Gene/transcript should not be *Adcyap1r1*. During production, PACAP peptide column was erroneously positioned under the PACAP receptor. The corrected Table 1 and legend appears below.

**Table 1:** Nomenclature for PACAP and its receptors.

**PACAP**	**PACAP Receptor**		
**Gene/transcripts**	**Peptide**	**Gene/transcripts**	**Protein**
**Primate**			
*ADCYAP1*	PACAP	*ADCYAP1R1*	PAC_1_
		*VIPR1*	VPAC_1_
		*VIPR2*	VPAC_2_
**Mammalian (non-primate)**			
*Adcyap1*	PACAP	*Adcyap1R1*	PAC_1_
		*Vipr1*	VPAC_1_
		*Vipr2*	VPAC_2_
**Aves**			
*ADCYAP1*	PACAP	*ADCYAP1R1*	PAC_1_
		*VIPR1*	VPAC_1_
		*VIPR2*	VPAC_2_
**Actinopterygii (non-teleost)**			
*adcyap1*	Pacap	*adcyap1r1*	Pac_1_
		*vipr1*	Vpac_1_
		*vipr2*	Vpac_2_
**Teleost**			
*adcyap1a*	Pacapa	*adcyap1r1a*	Pac_1_a
*adcyap1b*	Pacapb	*adcyap1r1b*	Pac_1_b
		*vipr1a*	Vpac_1_a
		*vipr1b*	Vpac_1_b
		*vipr2a*	Vpac_2_a
		*vipr2b*	Vpac_2_b
**Agnathan**			
*ADCYAP1*	PACAP	*VIPR*	VPAC
**Urochordate**			
*pacap1/pacap2*	PACAP	ni	ni
**Cephalochordate**			
*PACAP/GCG*	PACAP/GCG	*PACAP/GCGR*	PACAP/GCGR
**Protostomes**			
ni	PACAP	ni	ni
**Cnidaria**			
ni	PACAP	ni	ni

The authors apologize for this error and state that this does not change the scientific conclusions of the article in any way. The original article has been updated.

