# Supplementary figures and images for: Tracing the Origins of the Pituitary Adenylate-Cyclase Activating Polypeptide (PACAP)
Source: Front Neurosci. 2020 May 20;14:366. doi: 10.3389/fnins.2020.00366 (PMC7251081; doi:10.3389/fnins.2020.00366)

**A**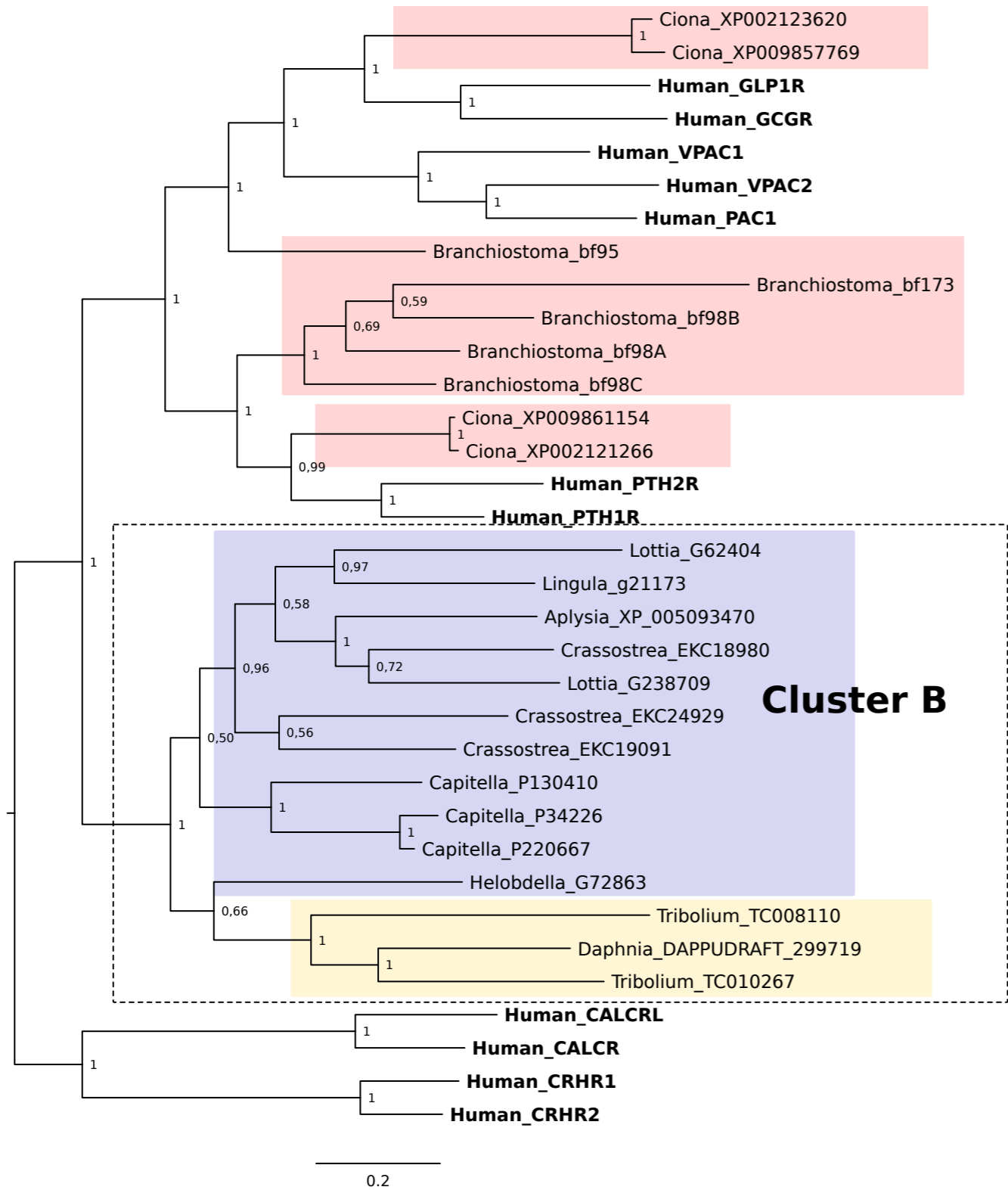**B**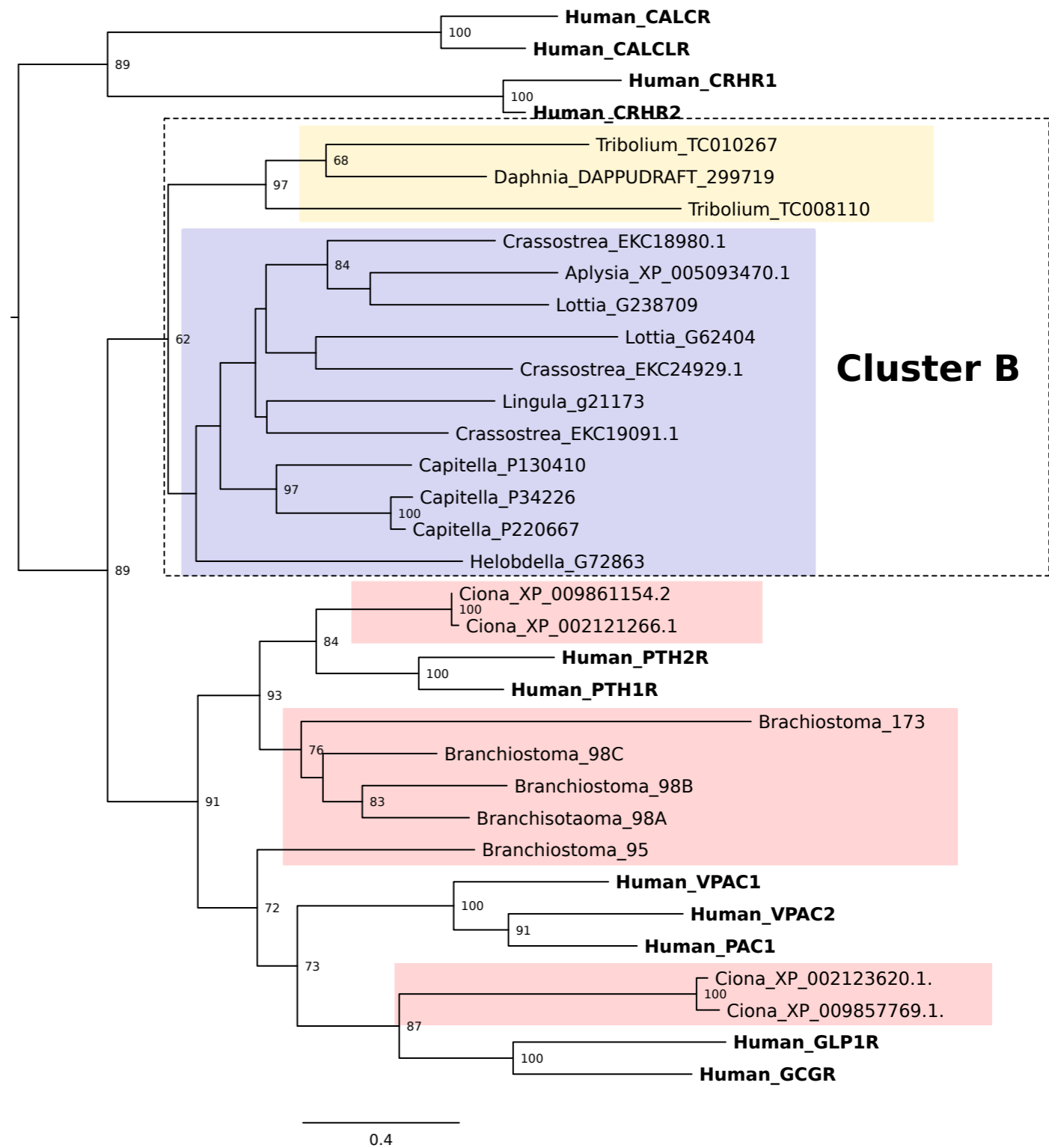

Supplement: FIGURE S1 — Phylogenetic trees of the invertebrate Cluster B receptors and the deuterostomes PAC1, GCGR, and PTHR members. The consensus phylogenetic trees were obtained (A) according to the Bayesian inference (BI) and (B) the maximum likelihood (ML) methods using has input an edited multiple sequence alignment of the predicted receptor protein sequences obtained using the MUSCLE algorithm (Edgar, 2004) available from Aliview platform 1.18 (Larsson, 2014). BI phylogenetic reconstruction was performed using MrBayes 3.2.6 (Ronquist et al., 2012) in the CIPRES Science Gateway V. 3.3 (http://www.phylo. org) and ML tree was performed using the PhyML3.0 program available from the ATGC platform (http://www.atgc-montpellier.fr/phyml/). The BI and ML trees were constructed with an LG substitution model according to the Akaike information criterion (Lefort et al., 2017) and BI used 1,000,000 generation sampling probability values to support tree branching and ML statistical branch support was 100 bootstrap replicates and >50 are mapped. The cephalochordate sequences are highlighted in pink and were obtained from On et al. (2015). The Arthropod receptor sequences are highlighted in yellow and were obtained from Cardoso et al. (2014) and the Lophotrochozoan sequences (highlighted in purple) were obtained by querying the metazoan ENSEMBLE GENOMES (http://metazoa. ensembl.org/index.html) database using the human receptors. Accession numbers of the invertebrate sequences are indicated in the tree. Accession numbers of human receptors are: CALCR, NP_001733.1; CALCRL, NP_005786.1; CRHR2, NP_001874.2; CRHR1, NP_004373.2; PTH1R, NP_000307.1; PTH2R, NP_005039.1; VPAC1, NP_004615.2; PAC1, NP_001109.2; VPAC2, NP_003373.2; GCGR, NP_000151.1; GLP1R, NP_002053.3. The tree was rooted using the human CALC/CALCRL and CRHR1/CRHR2 branches. [file Data_Sheet_1.PDF]
