# Supplementary material for: Tracing the Origins of the Pituitary Adenylate-Cyclase Activating Polypeptide (PACAP)
Source: Front Neurosci. 2020 May 20;14:366. doi: 10.3389/fnins.2020.00366 (PMC7251081; doi:10.3389/fnins.2020.00366)
Supplement: TABLE S3 — List of the hydra, protostome and tunicate PACAP nucleotide top five hits against the NCBI database (A) and Salmoniformes (taxid: 8006) transcriptomes (TSA) (B). The e-values (e value) and percent of identity (%ID) are given ∗ Nucleotide sequence not available. [file Table_3.DOCX]

**Supplementary Table 3**

**A)**

| **ADCYAP1 mRNA** | **TSA** | **WGS** | **E value** | **% ID** | **Hits (NCBI)** |
| --- | --- | --- | --- | --- | --- |
| **ECDYZOSOAN** | | | | | |
| *Periplaneta americana*  (AB083652)  (114 bp) | No significant similarities found | No significant similarities found | 4e-51  4e-51  4e-51  4e-51  4e-51 | 100%  100%  100%  100%  100% | XM_029700880.1- Salmo trutta glucagon family neuropeptides-like  XM_029626850.1-Oncorhynchus nerka glucagon family neuropeptides-like  LR584445.1- Salmo trutta genome assembly, chromosome: 2  XM_024374870.1- Oncorhynchus tshawytscha adenylate cyclase activating polypeptide 1  XM_024006657.1- Salvelinus alpinus glucagon family neuropeptides |
| *Eriocheir japonica*  AB121765.1  (114 bp) | Not available | Not available | 4e-51  2e-49  2e-49  2e-49  2e-49 | 100%  100%  100%  100%  100% | AB121765.1- Eriocheir japonica mRNA for pituitary adenylate cyclase activating polypeptide  XM_029756051.1- Salmo trutta glucagon family neuropeptides  XM_029661465.1- Oncorhynchus nerka glucagon family neuropeptides  LR584406.1- Salmo trutta genome assembly,  XM_024393020.1- Oncorhynchus tshawytscha glucagon family neuropeptides |
| *Litopenaeus vannamei** |  |  |  |  |  |
| **LOPHOCOTROZOA** | | | | | |
| *Sepioteuthis lessoniana*  (AB083651)  (114 bp) | Not available | Not available | 4e-51  4e-51  4e-51  4e-51  4e-51 | 100%  100%  100%  100%  100% | XM_029756051.1- Salmo trutta glucagon family neuropeptides  XM_029661465.1- Oncorhynchus nerka glucagon family neuropeptides  LR584406.1- Salmo trutta genome assembly  XM_024393020.1- Oncorhynchus tshawytscha glucagon family neuropeptides  XM_021562972.1- Oncorhynchus mykiss glucagon family neuropeptides |
| *Dugesia japonica*  (AB083649)  (114 bp) | No significant similarities found | No significant similarities found. | 4e-51  4e-51  4e-51  4e-51  4e-51 | 100%  100%  100%  100%  100% | XM_029756051.1- Salmo trutta glucagon family neuropeptides  XM_029661465.1- Oncorhynchus nerka glucagon family neuropeptides  LR584406.1- Salmo trutta genome assembly  XM_024393020.1- Oncorhynchus tshawytscha glucagon family neuropeptides  XM_021562972.1- Oncorhynchus mykiss glucagon family neuropeptides |
| **CNIDARIAN** | | | | | |
| *Hydra vulgaris*  (AB083650.1)  (114 bp) | No significant similarities found. | No significant similarities found. | 4e-51  4e-51  4e-51  4e-51  4e-51 | 100%  100%  100%  100%  100% | XM_029756051.1- Salmo trutta glucagon family neuropeptides  XM_029661465.1- Oncorhynchus nerka glucagon family neuropeptides  LR584406.1- Salmo trutta genome assembly  XM_024393020.1- Oncorhynchus tshawytscha glucagon family neuropeptides  XM_021562972.1- Oncorhynchus mykiss glucagon family neuropeptides |
| **INVERTEBRATE DEUTEROSTOME** | | | | | |
| *Halocynthia roretzi*  (AB121759.1)  (114 bp) | Not available | Not available | 4e-51  2e-49  2e-49  2e-49  2e-49 | 100%  100%  100%  100%  100% | [AB121759.1](https://www.ncbi.nlm.nih.gov/nucleotide/AB121759.1?report=genbank&log$=nuclalign&blast_rank=1&RID=02CX5M6X014)- Halocynthia roretzi mRNA for pituitary adenylate cyclase activating polypeptide  [XM_029756051.1](https://www.ncbi.nlm.nih.gov/nucleotide/XM_029756051.1?report=genbank&log$=nuclalign&blast_rank=2&RID=02CX5M6X014)- Salmo trutta glucagon family neuropeptides  XM_029661465.1- Oncorhynchus nerka glucagon family neuropeptides  LR584406.1- Salmo trutta genome assembly  XM_024393020.1- Oncorhynchus tshawytscha glucagon family neuropeptides |
| *Chelyosoma productum*  (507 bp) | Not available | Not available |  |  | No significant similarities found |
| *Chelyosoma productum*  (883-bp) | Not available | Not available |  |  | No significant similarities found |

**B)**

| **ADCYAP1 mRNA** | **E value** | **ID %** | **Salmoniformes**  **taxid:8006** | **Identity (NCBI)** |
| --- | --- | --- | --- | --- |
| *Periplaneta americana*  (AB083652)  (114 bp) | 5e-52  5e-52  5e-52  5e-52  5e-52 | 100%  100%  100%  100%  100% | EZ872471.1- O. mykiss  GGDU01301331.1- O. tshawytscha  GGDU01017810.1- O. tshawytscha  GGDU01017807.1- O. tshawytscha  GGDU01017804.1- O. tshawytscha | XM_014157815.1 - PREDICTED: S. salar glucagon family neuropeptides-like  XM_014157815.1 - PREDICTED: S.salar glucagon family neuropeptides-like  XM_014157815.1 - PREDICTED: S. salar glucagon family neuropeptides-like  XM_024374870.1 - PREDICTED: O. tshawytscha adenylate cyclase activating polypeptide 1  XM_014157815.1 - PREDICTED: S. salar glucagon family neuropeptides-like |
| *Sepioteuthis lessoniana*  (AB083651)  (114 bp) | 5e-52  5e-52  5e-52  5e-52  5e-52 | 100%  100%  100%  100%  100% | HAGJ01147357.1 - H. taimen  HAGJ01147355.1- H. taimen  GFIS01058303.1 - S. trutta  GFIN01062319.1 - O. mykiss  GFIN01062318.1 - O. mykiss | XM_014157816.1 - PREDICTED: Salmo salar glucagon family neuropeptides-like  XM_021619861.1 - PREDICTED: O. mykiss adenylate cyclase activating polypeptide 1  LR584406.1 – S. trutta genome assembly, chromosome: 6  NM_001139927.1 – S. salar Glucagon family neuropeptides (paca), mRNA  XM_021619858.1 - PREDICTED: O. mykiss adenylate cyclase activating polypeptide 1 |
| *Dugesia japonica*  (AB083649)  (114 bp) | 5e-52  5e-52  5e-52  5e-52  5e-52 | 100%  100%  100%  100%  100% | HAGJ01147357.1 – H. taimen  HAGJ01147355.1 – H. taimen  GFIS01058303.1- S. trutta  GFIN01062319.1 – O. mykiss  GFIN01062318.1-O. mykiss | XM_014157816.1 - PREDICTED: Salmo salar glucagon family neuropeptides-like  XM_021619861.1 - PREDICTED: Oncorhynchus mykiss adenylate cyclase activating polypeptide 1  [XM_014180582.1](https://www.ncbi.nlm.nih.gov/nucleotide/XM_014180582.1?report=genbank&log$=nucltop&blast_rank=3&RID=09NRDM5501R) - PREDICTED: Salmo salar Glucagon family neuropeptides (paca), transcript  XM_014180581.1 - PREDICTED: Salmo salar Glucagon family neuropeptides (paca),  LR664377.1 - Coregonus sp. 'balchen' genome |
| *Eriocheir japonica*  AB121765  (114 bp) | 2e-50  2e-50  2e-50  2e-50  2e-50 | 99.2%  99.2%  99.2%  99.2%  99.2% | HAGJ01147357.1- H. taimen  HAGJ01147355.1 - H. taimen  GFIS01058303.1 - S. trutta  GFIN01062319.1 - O. mykiss  GFIN01062318.1 - O. mykiss | XM_014157816.1 - PREDICTED: Salmo salar glucagon family neuropeptides-like  XM_021619861.1 - PREDICTED: Oncorhynchus mykiss adenylate cyclase activating polypeptide 1  [XM_014180582.1](https://www.ncbi.nlm.nih.gov/nucleotide/XM_014180582.1?report=genbank&log$=nucltop&blast_rank=3&RID=09NRDM5501R) - PREDICTED: Salmo salar Glucagon family neuropeptides (paca), transcript  XM_014180581.1 - PREDICTED: Salmo salar Glucagon family neuropeptides (paca),  LR664377.1 - Coregonus sp. 'balchen' genome |
| *Hydra vulgaris*  (AB083650)  (114 bp) | 5e-52  5e-52  5e-52  5e-52  5e-52 | 100%  100%  100%  100%  100% | HAGJ01147357.1- H. taimen  HAGJ01147355.1 - H. taimen  GFIS01058303.1 - S. trutta  GFIN01062319.1 - O. mykiss  GFIN01062318.1 - O. mykiss | XM_014157816.1 - PREDICTED: Salmo salar glucagon family neuropeptides-like  XM_021619861.1 - PREDICTED: Oncorhynchus mykiss adenylate cyclase activating polypeptide 1  [XM_014180582.1](https://www.ncbi.nlm.nih.gov/nucleotide/XM_014180582.1?report=genbank&log$=nucltop&blast_rank=3&RID=09NRDM5501R) - PREDICTED: Salmo salar Glucagon family neuropeptides (paca), transcript  XM_014180581.1 - PREDICTED: Salmo salar Glucagon family neuropeptides (paca),  LR664377.1-Coregonus sp. 'balchen' genome |
| *Halocynthia roretzi*  (AB121759)  (114 bp) | 2e-50  2e-50  2e-50  2e-50  2e-50 | 99.1%  99.1%  99.1%  100%  100% | HAGJ01147357.1- H. taimen  HAGJ01147355.1 - H. taimen  GFIS01058303.1 - S. trutta  GFIN01062319.1 - O. mykiss  GFIN01062318.1 - O. mykiss | XM_014157816.1 - PREDICTED: Salmo salar glucagon family neuropeptides-like  XM_021619861.1 - PREDICTED: Oncorhynchus mykiss adenylate cyclase activating polypeptide 1  [XM_014180582.1](https://www.ncbi.nlm.nih.gov/nucleotide/XM_014180582.1?report=genbank&log$=nucltop&blast_rank=3&RID=09NRDM5501R) - PREDICTED: Salmo salar Glucagon family neuropeptides (paca), transcript  XM_014180581.1 - PREDICTED: Salmo salar Glucagon family neuropeptides (paca),  LR664377.1-Coregonus sp. 'balchen' genome |
| *Chelyosoma productum* |  |  | No significant similarities found |  |
